# Acupotomy for shoulder pain: A protocol for systematic review

**DOI:** 10.1097/MD.0000000000025502

**Published:** 2021-04-16

**Authors:** Yongda Zhu, Yiwen Zhu, Renying Ye, Danyun Hua, Cimin Shen

**Affiliations:** a Medical Community of Fenghua District Hospital of Traditional Chinese Medicine of Ningbo, Ningbo; b Zhejiang Chinese Medical University, Grade Three, Traditional Chinese Medicine, Hangzhou, Zhejiang, China.

**Keywords:** acupotomy, protocol, shoulder pain, systematic review

## Abstract

**Background:**

Shoulder pain is a common musculoskeletal disorder prompting many patients to seek treatment. Acupotomy is a common treatment for shoulder which has been widely used in hospitals. But its efficiency has not been scientifically and methodically evaluated. This protocol aims to evaluate the efficacy and safety of acupotomy for treating shoulder pain.

**Methods:**

Relevant studies will be searched from the databases of PubMed, EMBASE, Cochrane Library, China Knowledge Resource Integrated Database, Weipu Database for Chinese Technical Periodicals, SinoMed, and Wanfang Database. Two researchers will independently select studies, collect data, and assess the methodology quality by the Cochrane risk of bias tool.

**Results:**

The systematic review will provide high-quality evidence to assess the efficacy and safety of acupotomy for shoulder pain as well as adverse events.

**Conclusion:**

The systematic review will provide evidence to assess the effectiveness and safety of acupotomy therapy for shoulder pain patients.

**INPLASY registration number:**

INPLASY 202130002.

## 1 Introduction

Shoulder pain is a common musculoskeletal disorder with increase prevalence from 7.5% to 21%.^[1–3]^ According to the diagnosis of shoulder pain, it involves adhesive capsulitis, rotator cuff tendinitis, shoulder impingement syndrome, acromioclavicular joint disease, and so on.^[1]^ Patients with shoulder pain usually suffer from pain, active and passive motion restriction, and function limitation.^[4]^ So shoulder pain is the main cause of sick leave in western countries.^[5,6]^ In China, the incidence of women over 40 is 45%, and the incidence is 8% for urban population.^[7]^ In traditional Chinese medicine (TCM), according to the theory of TCM, shoulder pain always knows as frozen shoulder. At present, for shoulder pain patients, the aim of treatments mainly focus on restoration of shoulder joint function and reduction of pain intensity.^[8]^

There are many related conservation treatment applied on shoulder pain, such as corticosteroids, nonsteroidal anti-inflammatory drugs, acupuncture, manual therapy, and other physical therapy. And the effectiveness of most intervention is not definite.^[9]^ Acupotomy is a new-style bladed needle including flathead and cylindrical body. According to the theory of acupotomy, the therapy combined traditional Chinese acupuncture and modern surgery principle. It evolved from acupuncture needle.^[10]^ It has been widely used in TCM hospital for many disease.^[11]^ And the efficacy of acupotomy for knee osteoarthritis has been verified.^[12–14]^ However, at present, no systematic review reported on the efficacy and safety of acupotomy for shoulder pain. Therefore, the systematic review will assess the efficacy and safety of acupotomy for shoulder pain.

## 2 Methods

This systematic review protocol has been registered on INPLASY (https://inplasy.com/inplasy-2021-3-0002/). The registration number is INPLASY 202130002. This protocol was performed in accordance with the preferred reporting items for systematic reviews and meta-analysis protocol. Ethical approval is unnecessary because this is a literature-based study.

### 2.1 Inclusion criteria for study selection

#### 2.1.1 Type of studies

All randomized controlled trials (RCTs) of acupotomy therapy for shoulder pain will be included without publication status restriction or writing language. Designs such as animal experiments, reviews, quasi-RCTs, case reports, and laboratory studies will be rejected.

#### 2.1.2 Types of patients

People with shoulder pain regardless of sex, age, or severity and duration of disease.

#### 2.1.3 Type of interventions

The experimental group will be treated with acupotomy. There are no limitation of the needle materials, and methods. False acupotomy is not reported in literature. The acupotomy therapy is used widely in the Chinese hospitals. The control group will adopt the therapy such as block, placebo, or no treatment. Trials that evaluate acupotomy as a combination to other therapies will also be included.

#### 2.1.4 Types of outcomes

The primary outcome of shoulder pain symptom is visual analog scale (0–10), the ability assessment of daily living activities. Adverse events incidence and shoulder range of motion will be accepted as the secondary outcomes.

### 2.2 Search methods for identification of studies

#### 2.2.1 Electronic searches

Relevant studies will be searched in the following electronic databases: Pubmed, EMBASE, Cochrane Library, China Knowledge Resource Integrated Database, Weipu Database for Chinese Technical Periodicals, Sinomed, and Wanfang Database. The search terms include small needle knife, acupotomy, needle knife, shoulder impingement syndrome, rotator cuff, bursitis, adhesive capsulitis, frozen shoulder, shoulder pain, and RCTs. The equivalent search words will be used in Chinese databases. All search terms are included in Table 1, and other searches will be based on these results.

**Table 1:** Search strategy used in PubMed.

Search	Search terms
1	((((shoulder impingement syndrome) OR rotator cuff) OR bursitis) OR adhesive capsulitis) OR frozen shoulder) OR shoulder pain
2	((acupotomy) OR small needle-knife) OR needle knife
3	(((random[Text Word] OR randomized[Text Word]) OR control[Text Word]) OR controlment[Text Word]) OR trial[Text Word] AND “humans”[MeSH Terms]
4	#1 AND #2AND #3

#### 2.2.2 Searching other resource

Additionally, we will search the international clinical trials registry platform, dissertation, and gray literature to identify studies related to acupotomy for shoulder pain. The relevant conference papers, journals will be retrieved manually.

### 2.3 Data collection and analysis

#### 2.3.1 Selection of studies

**Figure 1:**
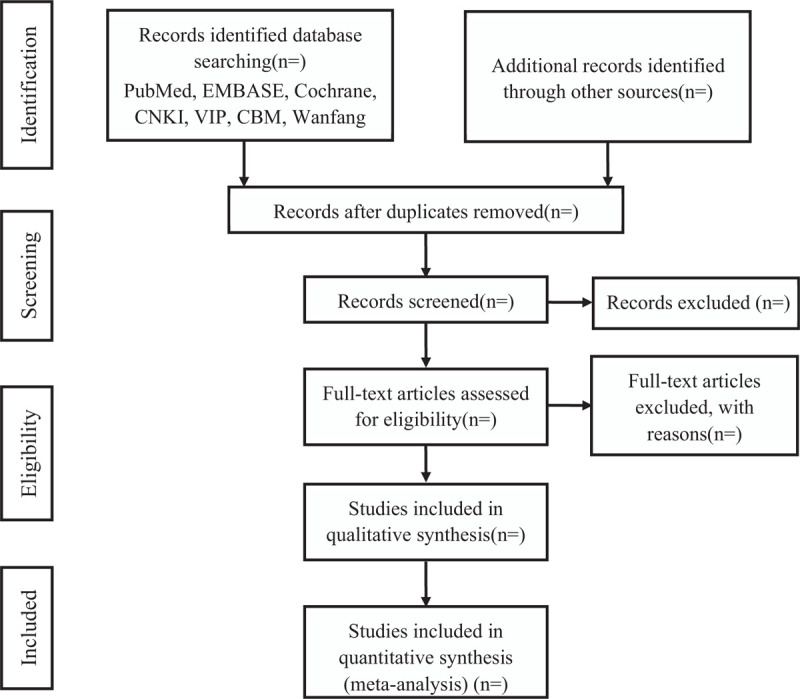
The PRISMA flow diagram of study selection process. PRISMA = preferred reporting items for systematic review and meta-analysis.

Two researchers will independently discuss and determine research selection process according to the criteria. All literatures will be imported to the endnote X9. We will remove the duplicated data and screen records by title and abstract and the full article. Any study excluded should be labeled on full article. If there is difference in the research choices, we will resolve it by discussing with the third author. Screening study flow diagram is summarized as Figure 1.

#### 2.3.2 Data extraction and management

A standard form will be designed for data collection first of all. Two researchers will independently extract data of studies and record on the form. For the ambiguity of studies, it will be solved by expert discussion. We will contact authors for more information when necessary. The extracted data contain the first author, publication year, participants characteristics, interventions, duration of treatment, follow-up, outcome assessment, research results, adverse events, and other detailed information. If any details of the article are incomplete, we will contact the appropriate author for more information.

#### 2.3.3 Assessment of risk of bias

Two researchers will independently evaluate the risk and bias using the Cochrane collaboration's tool. These items included in this toll will be evaluated: random sequence generation, allocation concealment, the blinding method for patients, researchers and outcomes assessors, incomplete outcome data, and selective reports. The bias risk for every item will be classed as “low risk of bias,” “high risk of bias,” “unclear risk of bias.”

#### 2.3.4 Measures of treatment effect

For dichotomous data, risk ratio with 95% confidence interval (95% CIs index) will be used to evaluate the effect. For continuous data, standard mean difference or mean difference with 95% CI will be presented.

#### 2.3.5 Missing data management

For some articles, if there is incomplete data, we will try to contact the first or corresponding author by email. If the missing data is not available, we will analyze the data acquired.

#### 2.3.6 Assessment of heterogeneity

The research will be performed by Review Manager Version 5.3 software. Heterogeneity will be evaluated by chi-squared test. If *I*^2^ value is less than 50%, indicating significant heterogeneity statistical results, we will use random effects model. If not, the fixed effects model, standardized mean difference, and corresponding 95% CIs will be applied for further data.

#### 2.3.7 Subgroup analysis

If there is a significant heterogeneity in the studies, we will perform a subgroup analysis based on the type of acupotomy, treatment duration, different controls, and outcome measurements.

#### 2.3.8 Data synthesis

All data will be combined and analyzed with the Cochrane Collaboration software (Review Manager Version 5.3 for Windows; Copenhagen: The Nordic Cochrane Centre). If *I*^2^ value is less than 50%, fixed effect model will be selected to pool data. Otherwise, we will use the random effect model to synthesize data. And the subgroup analysis will be performed. If the clinical heterogeneity still is obvious, we will not be able to implement the meta-analysis, only descriptive analysis instead.

#### 2.3.9 Sensitivity analysis

When there are sufficient studies, we will carry out sensitivity analysis to test the robustness of studies according to the quality of method, the sample size, and the selection of missing data. And the fluctuation of results will be observed.

#### 2.3.10 Reporting bias

If there are enough studies include (more than 10), funnel plot will be performed. And the Egger regression and the Begger tests will be calculated to check the asymmetry of funnel plot.

## 3 Discussion

Shoulder pain is one of the most common orthopedic diseases, which affect about 20% to 30% of the general population.^[15]^ So this disease bring economic, physical, and psychological burdens to people in many countries. At present, pharmacologic and nonpharmacologic treatments are the main choice for this disease. Acupotomy is a new therapy from TCM. And this therapy is a miniature surgery with less pain.^[16]^ But up to now, there is no systematic evaluation reported regarding the therapeutic effectiveness and safety for shoulder patients.

Therefore, the purpose of this proposed systematic review is to assess the efficacy and safety of acupotomy for shoulder pain. This study may have some limitations because of the imperfect data collection such as unpublished data. And language and different measurements and tools may lead to the risk of heterogeneity. We expect to have an objective, true and high quality evaluation of the studies on clinical trials of acupotomy for shoulder pain.

## Author contributions

**Conceptualization:** Yongda Zhu, Yiwen Zhu.

**Data curation:** Danyun Hua, Cimin Shen.

**Formal analysis:** Danyun Hua, Cimin Shen.

**Methodology:** Renying Ye, Danyun Hua, Cimin Shen.

**Project administration:** Renying Ye.

**Supervision:** Yiwen Zhu, Renying Ye.

**Writing – original draft:** Yongda Zhu.

**Writing – review & editing:** Yongda Zhu, Yiwen Zhu.
